# Using Nuclear Magnetic Resonance to Troubleshoot a Stability Issue in a Real-World Formulation Chassis—Application to Consumer Oral Healthcare

**DOI:** 10.3390/pharmaceutics16030320

**Published:** 2024-02-24

**Authors:** Tomris Coban, Hannah Sykes, Shreedhar Kulkarni, Robert A. Lucas, Cameron Robertson, Adam Le Gresley

**Affiliations:** 1 Life Sciences, Pharmacy and Chemistry, HSSCE Faculty, Kingston University, Kingston upon Thames KT1 2EE, UK; t.coban@kingston.ac.uk (T.C.); c.robertson@kingston.ac.uk (C.R.); 2 Haleon PLC, Weybridge KT13 0DE, UK; hannah.i.sykes@haleon.com (H.S.); shreedhar.x.kulkarni@haleon.com (S.K.); robert.a.lucas@haleon.com (R.A.L.)

**Keywords:** salicylic acid, NMR, formulation, discoloration

## Abstract

With direct application to current and future consumer healthcare products, this research sheds light on the importance of packaging and its potential effects on both Active Pharmaceutical Ingredient (API) delivery and stability. Industrially sourced, proprietary experimental formulations (PEFs), specifically oral cleansers, based on salicylic acid and hydrogen peroxide, discolored over time at different rates, depending on packaging type used. This discoloration stemmed from an interplay of two factors, involving both spontaneous formulation degradation and the interaction of both degradants and salicylic acid with the internal surface of the packaging. This manuscript reports on the investigation to uncover the origins of discoloration. To investigate this real-world, industrial pipeline problem, we exploited the high dimensionality and simple sample preparation uniquely afforded by NMR. Using a combination of 1D/2D NMR and diffusion-ordered NMR spectroscopy (DOSY) to leverage molecular mass estimations from, we not only quickly confirmed the identities of these degradants, but also assessed their formation as a function of temperature and pH, providing insight into the mechanisms underlying their formation. We were able to identify catechol as the main source of discoloration over a period of several weeks, being formed at the ppm level. Furthermore, we evaluated the formulation–container interaction, employing NMR, ICP-MS, and ATR-IR. Despite this comprehensive analysis, the root causes of discoloration could only tentatively be assigned to a surface Ti complex of salicylic acid and other hydroxy carboxylic acids. Through the understanding of formulation degradation pathways, we were able to support further toxicology assessment, vital to both consumer safety and the manufacturer. This work underscores the invaluable role of NMR in the analysis of intricate proprietary mixtures with a consumer-centric purpose. Our findings demonstrate that conventional analytical techniques falter in the face of such complexity, requiring extensive preparation and pre-analytical processing, highlighting the novelty and crucial relevance of NMR research to manufacturers and consumers. Such an analysis is of value in the pursuit of materials within the consumer-healthcare space, which meet the requirements for successful recycling or re-use.

## 1. Introduction

Oral health products, such as toothpaste, mouthwash, denture cleaners, and dental gels, are essential components of daily oral hygiene routines for millions of people worldwide. These products typically contain active ingredients, such as fluoride, antimicrobial agents, and desensitizing agents, that play crucial roles in preventing dental diseases and maintaining oral health [1]. However, one of the significant challenges faced by manufacturers and consumers of these products is the degradation of active components over time, leading to reduced efficacy and potential safety concerns [2]. Active components in oral health products are susceptible to various degradation mechanisms, including chemical degradation, physical instability, and microbial degradation. Chemical degradation, such as hydrolysis, oxidation, and photodegradation, can occur due to exposure to moisture, oxygen, light, and temperature fluctuations during storage and use [3]. Physical instability, such as phase separation, particle aggregation, and crystal growth, can compromise the uniform distribution and bioavailability of active components in formulations [4]. Microbial degradation, resulting from contamination with bacteria and fungi, can lead to spoilage and the deterioration of product quality [5]. For example, fluoride, a widely used active ingredient in toothpaste for its caries-preventive properties, can degrade over time due to interactions with other ingredients, exposure to air and moisture, and inadequate packaging [6]. This degradation can result in reduced fluoride levels in the product, compromising its effectiveness in preventing tooth decay [7]. Analyzing and troubleshooting degradation issues in oral health products are crucial for several reasons:

- Maintaining Efficacy: Active components play a pivotal role in the efficacy of oral health products. Understanding and mitigating degradation pathways ensure that products deliver the intended benefits to consumers, such as cavity prevention, plaque reduction, and gum disease management [8].
- Regulatory Compliance: Regulatory agencies, such as the Food and Drug Administration (FDA) in the United States and the European Medicines Agency (EMA) in Europe, impose strict guidelines on the quality, stability, and safety of oral health products [9]. Failure to address degradation issues may lead to non-compliance with regulatory requirements and potential product recalls.
- Protecting Brand Reputation: Product quality and consistency are essential for maintaining consumer trust and brand reputation [10]. Addressing degradation issues promptly demonstrates a commitment to product excellence and customer satisfaction [11].

Many of the above issues are dealt with “in house” and are often the subject of internal reports rather than peer-reviewed discussion. It is for this reason and to espouse the value of a detailed analysis to formulation development that this work is presented. The degradation of active components in consumer oral health products presents significant challenges that impact product efficacy, safety, regulatory compliance, and brand reputation [12]. Analyzing and troubleshooting degradation issues are essential for ensuring product quality, safety, and effectiveness, ultimately contributing to improved oral health outcomes for consumers [13,14].

### 1.1. NMR Techniques

Nuclear magnetic resonance (NMR) techniques have firmly established themselves as invaluable tools for the assessment of component degradation and the exploration of physical interactions within pharmaceutical formulations [15,16,17]. Although NMR is considered less sensitive compared to techniques like LC-MS or HPLC, it compensates with its exceptionally high resolution and dimensionality. One of its notable advantages lies in its ability to analyze concentrated samples quantitatively without the necessity for extensive separation steps [18].

Diffusion ordered spectroscopy (DOSY) NMR relies on the inherent property of molecules to diffuse through a solution at different rates. The rate of diffusion is primarily determined by the size and shape of the molecule. In DOSY NMR, a series of NMR spectra are collected at different gradient strengths, which induce a gradient of the magnetic field. The gradient strength affects the precession frequency of nuclei in the sample, allowing for the differentiation of species based on their diffusion coefficients. By analyzing the data from these spectra, it is possible to construct a diffusion profile, which reveals the individual contributions of each species present in the sample [19].

DOSY NMR, in particular, emerges as a powerful technique for delving into the physical interactions between components, often eluding detection by many mass spectrometry (MS) techniques [18]. DOSY’s unique capability to differentiate components based on their hydrodynamic radius adds an extra layer of sophistication, enhancing discrimination in pharmaceutical and industrial processes [20]. This ability to “filter” components based on their size is especially advantageous in complex formulations, where various molecular species may coexist.

In the context of this research, proprietary experimental formulations (PEFs) featuring hydrogen peroxide, salicylic acid (SA), Sodium dodecyl sulphate (SLS), an acidity regulator, and proprietary flavoring were evaluated at different temperatures over various time periods to better understand the pathways to degradation. To augment the rigor of this study, experimental formulations were also prepared to further explore the apparent mechanisms. The choice of these components reflects a deliberate selection to simulate real-world industrial formulations, where the interplay of multiple ingredients is both intricate and crucial.

The utilization of NMR techniques, including DOSY, in this study serves as a testament to their efficacy in addressing the challenges posed by complex formulations. While MS techniques may offer higher sensitivity, the unparalleled resolution and discrimination capabilities of NMR, especially in probing physical interactions, make it an indispensable tool in pharmaceutical and industrial research [18].

By exploiting the advantages of NMR, this research not only contributes to the understanding of degradation pathways but also sheds light on the nuances of physical interactions between components. In an industry where the quality and stability of formulations are paramount, the insights gleaned from this study hold promise for refining manufacturing processes and ensuring the efficacy and safety of consumer healthcare products.

### 1.2. Rationale and Observations

This research was initiated by an observation by Haleon PLC (Weybridge, UK) that certain packaged experimental denture cleanser formulations were discoloring over a 2-month period when stored at 25–40 °C. Whilst degradation to conjugated/complexed compounds was likely the cause of discoloration, there appeared to be two different processes occurring, depending on the composition of the container (HDPE (White), HDPE (Natural), PET, and glass).

**Figure 1:**
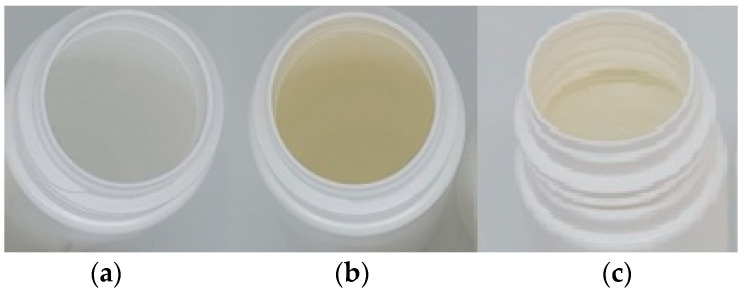
Full proprietary formulation stored in HDPE (White). (**a**) Formulation and container at 0 days is White. (**b**) Formulation after 6 weeks at 40 °C. (**c**) Container discoloration after 6 weeks at 40 °C.

The yellowing of the internal packaging was taking place over 6 weeks at 40 °C and occurred only with HDPE (White). However, for all container types, the formulation itself developed a distinctive orange appearance over a 6-week to 2-month timeframe, when stored at 25–40 °C. A representative example is shown in Figure 1. The obvious hypothesis was that formulation degradation over time would lead to the direct adsorption of colored degradants onto the internal surfaces of the container, but only HDPE (White) showed this persistent container yellowing, suggesting that passive adsorption was not the cause. Hence, the authors separated the two processes into those associated with packaging discoloration and those associated with formulation discoloration.

#### 1.2.1. Packaging Discoloration

Packaging discoloration intensified over time and at elevated temperatures used to simulate long-term storage (Figure 1). Notably, HDPE (Natural), PET, and glass containers showed no internal surface discoloration even after 6 weeks at 40 °C. The discoloration was specific to HDPE (White), the composition differing only in the presence of TiO_2_ (50%) in the HDPE (White) polymer mix.

#### 1.2.2. Formulation Discoloration

Formulation discoloration occurred regardless of container type. Discoloration appeared within 2 months, even in glass at 25 °C, and became more pronounced at 40 °C and 50 °C used to accelerate degradation for stability measurement. The objective was to identify the cause(s) of discoloration within the proprietary experimental formulations (PEFs) and establish a pathway for their formation. Once identified, these causative agents could be quantified to assess their toxicological impact.

## 2. Materials and Methods

Proprietary formulation samples were received from Haleon PLC (Weybridge, UK) after temperature (°C)- and humidity (psf)-dependent treatment (25/60, 40/75, 50/75) in different packaging types (HDPE (White), HDPE (Natural), PET, and glass). Samples were provided in 50 mL glass jars or 500 mL HDPE/PET bottles with 0.63 mL aliquoted from each to prepare NMR samples. Samples consisted of a base chassis of salicylic acid (Merck Life Sciences, Gillingham, UK) and H_2_O_2_ (Merck Life Sciences, Gillingham, UK) with specific excipients added and removed to evaluate the effect on the formulation stability and salicylic acid state.

The reference numbers for the samples are in Supporting Information (Table S1), indicating the specific formulation sub-set conditions, packaging, and additions that were evaluated for stability effects. Samples were submitted for NMR experiments that quantitatively monitored the chemical composition relative to thermal variability, after total assignment of all detected compounds in formulations.

### 2.1. PEFs

Samples, unless specified otherwise, were provided by Haleon PLC (Weybridge, Unied Kingdom). Samples consisted of a base chassis of 0.3% *w*/*v* glycerol base flavoring Optamint^TM^, 0.18% salicylic acid (Merck Life Sciences, Gillingham, UK) and 1.3% H_2_O_2_ (30% (*w*/*w*) in H_2_O, containing stabiliser (Merck Life Sciences, Gillingham, UK)), and 0.5% SLS (Merck Life Sciences, Gillingham, UK). Conditions, packaging, and time frame of the analysis are included in Table S1. Samples were made up in different packaging types and conditions infarcted upon them at Haleon PLC (Weybridge, UK) labs and delivered to Kingston for the analysis at times detailed in Table S1. Samples were delivered in packaging that experiments took place in on the day that experiments finished. Upon receipt at Kingston University, samples were logged, with receipt and production dates recorded. Samples not prepared and analyzed by NMR on the day of delivery were refrigerated and sealed to minimize changes in formulation composition. The evaluation of differences between refrigerated and immediately analyzed samples is presented in Table S2.

Samples were transferred directly to NMR tubes (Wilmad 7’ 5 mm high precision) (Merck Life Sciences, Gillingham, UK) (0.63 mL) using 1 mL sterile pipette tips (Merck Life Sciences, Gillingham, UK) with a Pipet-Lite LTS Pipette L-2000XLS+ (Mettler Toledo, Leicester, UK) in a fume hood to minimize the potential contamination of samples. Unopened glass vials of D_2_O with an internal reference TSP (99.9 atom% D, 0.05 wt.% 3-(trimethylsilyl)propionic-2,2,3,3-d4 acid, sodium salt) (Merck Life Sciences, Gillingham, UK) were added to NMR tubes (Wilmad 7’ 5 mm high precision) (Merck Life Sciences, Gillingham, UK) (0.07 mL). A maximum of 1 h was allowed before samples were analyzed via NMR to allow for interleaved triplicated experiments to be run between samples.

ATR-IR and inductively coupled plasma optical emission spectroscopy (ICP-OES) were used for the surface analysis of packaging and elemental analysis, respectively. ICP-OES samples were prepared with the addition of nitric acid (70%) to make 2% nitric acid, to the solution and to different packaging types. Packaging acid preparations were agitated for 24 h before the ICP-OES analysis, with solutions being immediately analyzed after acidification. The ATR-IR analysis was conducted on packaging tokens (2 cm^2^) that were cut out of emptied and dried packaging, with the internal surface being analyzed. This was conducted for HDPE (White), HDPE (Natural), and PET packaging of formulation bottles.

### 2.2. Model Samples

In-house sample preparation for further condition evaluation and validation of specific steps in the process followed proprietary sample chassis composition as detailed in Section 2.1. Amounts were determined based on proprietary formulation specifications, and pH adjustment was performed as the final step using 10% NaOH/10% HCl (Merck Life Sciences, Gillingham, UK) with pH monitored using a pH meter (Thermoscientific Orion 2—Thermofisher Scientific PLC, Hemel Hempstead, UK).

To model the effect of pH on the series of oxidation reactions responsible for degradant formation, a range of simplified solutions comprising 0.5% H_2_O_2_, 0.18% salicylic acid, 0.5% SLS, and 10% NaOH were added at varying levels to give a range of pH between 2 and 9. These were subsequently prepared for NMR as set out in Section 2.1.

### 2.3. NMR

All samples contained 10% D_2_O (99.9 atom % D, contains 0.05 wt.% 3-(trimethylsilyl)propionic-2,2,3,3-d4 acid, sodium salt) (Merck Life Sciences, Gillingham, UK) to provide a lock signal for NMR and maintain an in situ aqueous environment while serving as a chemical shift and diffusion reference. Fresh NMR tubes (Wilmad 7’ 5 mm high precision) (Merck Life Sciences, Gillingham, UK) were used for each sample to ensure consistency and minimize contamination with common cleaning solvents.

A range of NMR experiments, including ^1^H 1D, ^1^H ^13^C HSQC, ^1^H-^1^H TOCSY, and ^1^H 2D DOSY NMR, were conducted on a Bruker Avance III 600 MHz NMR (Bruker UK Ltd., Coventry, UK) with a TXI probe. Standard acquisition parameters for experiments included 1D ^1^H: noesygppr1d P1 (90-degree flip angle pulse) = 7 μs, flip angle = 90°, D1 (relaxation delay) = 10 s, NS = 64, O1P = 4.7 ppm, D8 (mixing time) = 0.01 s, PLW9 (low power pulse) = 7.2567 × 10^−5^ W during the relaxation delay and mixing time for the appropriate saturation of water peaks. Two-dimensional experiments included ^1^H-^13^C HSQC using pulse hsqcedetgpsisp2.4, a 2D H-1/X correlation via double inept transfer with NS = 32, D1 = 1.5 s; ^1^H-^1^H TOCSY using pulse dipsi2esgpph, NS = 16; 2D ^1^H DOSY; pulse sequence ledbggppr2s with a linear pulsed field gradient over 32 steps, NS = 16, P30 = 2000 µs, D20 = 0.1 s. All NMR processing was undertaken using Topspin 4.2 (Bruker UK Ltd., Coventry, UK)

### 2.4. ATR-IR and ICP-MS

ATR-IR was conducted on a Thermo Scientific^TM^iD5 ATR accessory for a Nicolet IS 5 spectrometer (East Grinstead, UK) with absorbance measured over wavenumbers 450–1750 cm^−1^. ICP-MS (Agilent 7700 Series/ASX-500 Series Autosampler) (Agilent Technologies, Wokingham, UK) was conducted with standard ICP and helium gas ICP to distinguish polymeric species that may be present within the solutions. Standard 2% NO_2_ blanks and sample 2% NO_2_ blanks were run alongside 0–100 ppm elemental reference solutions to give ppm calibration for ICP-MS data.

## 3. Results and Discussion

With it being apparent that there were potentially two mechanisms operating to discolor either the packaging or the formulation, the results are considered separately below.

### 3.1. Formulation Discoloration

#### 3.1.1. Initial Steps

**Figure 2:**
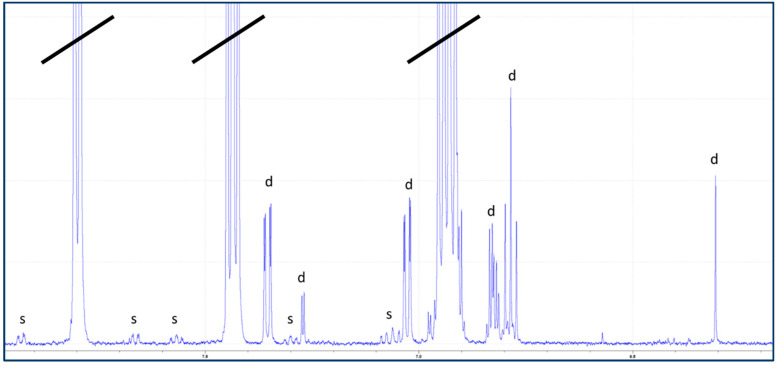
^1^H NMR spectrum (zoomed) of proprietary formulation in glass container 7 weeks at 40 °C, indicating appearance of signals correlating to carbon satellites of salicylic acid (s) and signals belonging to degradants (d). Black lines indicate high intensity salicylic acid peaks that extend past the spectral window.

The initial characterization of degraded vs. non-degraded PEFs was achieved using a mixture of 1D and 2D NMR techniques. This enabled the identification of expected components and degradants maleic acid, catechol, 2,3-dihydroxybenzoic acid (2,3-DHBA) (Figure 2).

**Figure 3:**
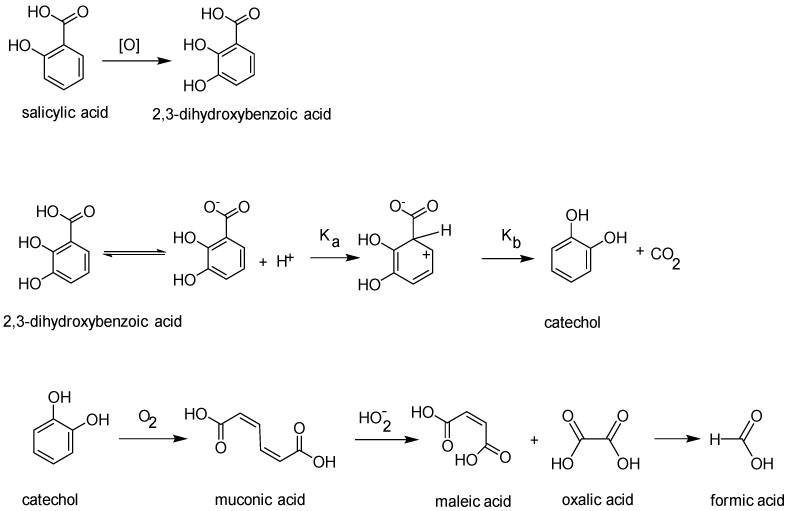
Mechanism for the proposed in situ salicylic acid oxidation steps that took place within the bulk formulation, yielding inherently colored catechol and illustrating potential source of decolorization of formulation within proprietary samples [21].

The oxidation of salicylic acid by hydrogen peroxide is not without precedent and the mechanism (Figure 3) indicates how the identified degradants, including formic acid, may be formed [5,11]. For comparison, ^1^H NMR spectra of pure salicylic acid are shown in Supporting Information Figure S1 to indicate the presence of ^13^C satellites when zoomed in.

**Figure 4:**
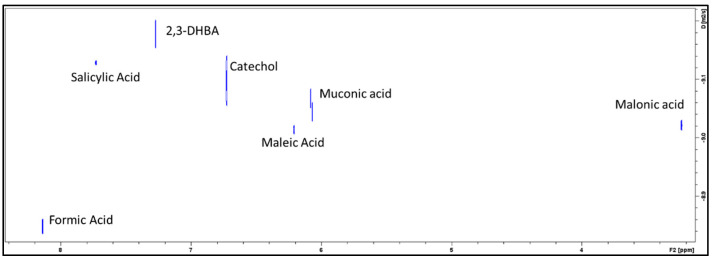

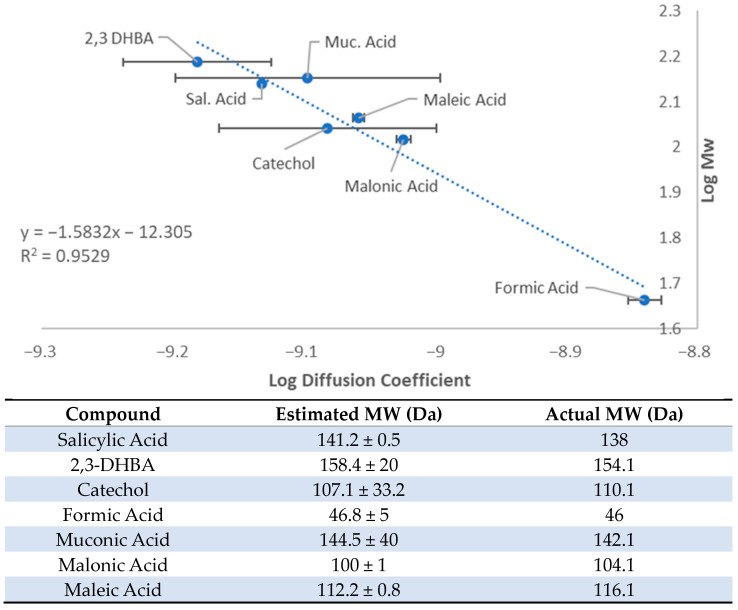
(**Top**) Two-dimensional DOSY spectrum for proprietary formulation in glass container at 7 weeks at 40 °C. (**Middle**) Degradants tentatively assigned based on diffusion to predict MW. Internal diffusion standards were trimethylsilylpropionate (TSP), formic acid, and salicylic acid. All diffusion values were referenced to TSP [25,26,27]. (**Bottom**) DOSY-estimated MW vs. Actual MW for observed compounds.

To validate the characterization of the degradants, DOSY spectra were used to generate an MW vs. LogD calibration curve to determine the approximate MW of the assigned degradant peaks (Figure 4). Combining the observed ^1^H signals with the DOSY spectra enabled the identification of the degradants [22,23,24] (Figure 4).

A further validation of this observation was achieved through control sample spiking (see Supporting Information Figure S2). It was quite apparent that the oxidation product responsible for the yellowing over time was catechol and the increase in its concentration over time correlated to the discoloration of the solution in all packaging. It is also worthy to note that decarboxylation only occurs from 2,3-DHBA. There was no NMR evidence of phenol being formed from the direct decarboxylation of SA, nor were quinone or hydroquinone degradants of the SA.

#### 3.1.2. Impact of Packaging on Rate of Formulation Discoloration

**Figure 5:**
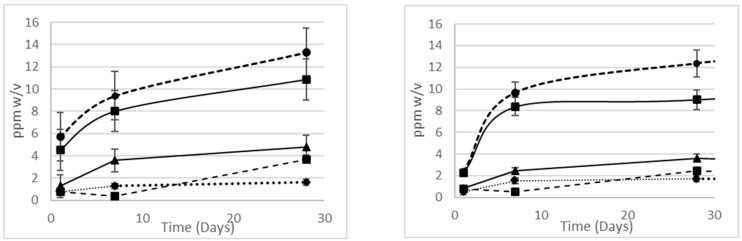
Evolution of degradants over time for PEF (pH 3) stored in (**left**) HDPE (White) and (**right**) PET containers: formic acid (-●-), maleic acid (■), catechol (▲), muconic acid (▪ ▪◆▪ ▪), 2,3 DHBA (-■-).

Concentrations of the degradants over time, stored in the different packaging material, are shown below (Figure 5) and there is no statistically significant difference for the evolution of the catechol, which is largely responsible for the discoloration of the PEF. The rate of maleic and formic acid formation is greater for PET than HDPE. The comparison of catechol formation in the PEF for both HDPE and PET with the same in glass packaging showed no significant difference, supporting the hypothesis that the discoloration of the PEF over time is driven directly by the oxidation of SA.

#### 3.1.3. Temperature Effects

**Figure 6:**
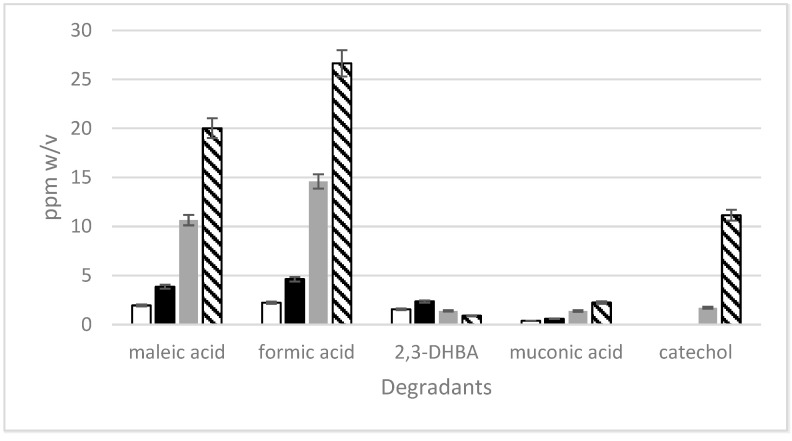
Evolution of degradants from the proprietary formulation stored in glass as a function of temperature over 12-week storage at temperatures 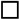 15 °C, 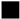 25 °C, 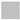 40 °C, 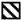 50 °C.

The PEF was stored in glass and sampled over a protracted period to gauge the effects of temperature on the evolution of the degradants. Unsurprisingly, elevated temperatures correlate positively to degradant formation, and temperature acceleration is used routinely for stability determination. Interestingly, the kinetics of the catechol forming reaction oxidation reaction, which is the main cause of the formulation discoloration, are significantly more retarded at lower temperatures, when compared to other oxidative degradation pathways, implying a substantially higher activation energy for the decarboxylation step. Catechol is observed at RT, but over a substantially longer timeframe (Figure 6).

#### 3.1.4. pH Effects on Degradation in Simplified Solutions

**Figure 7:**
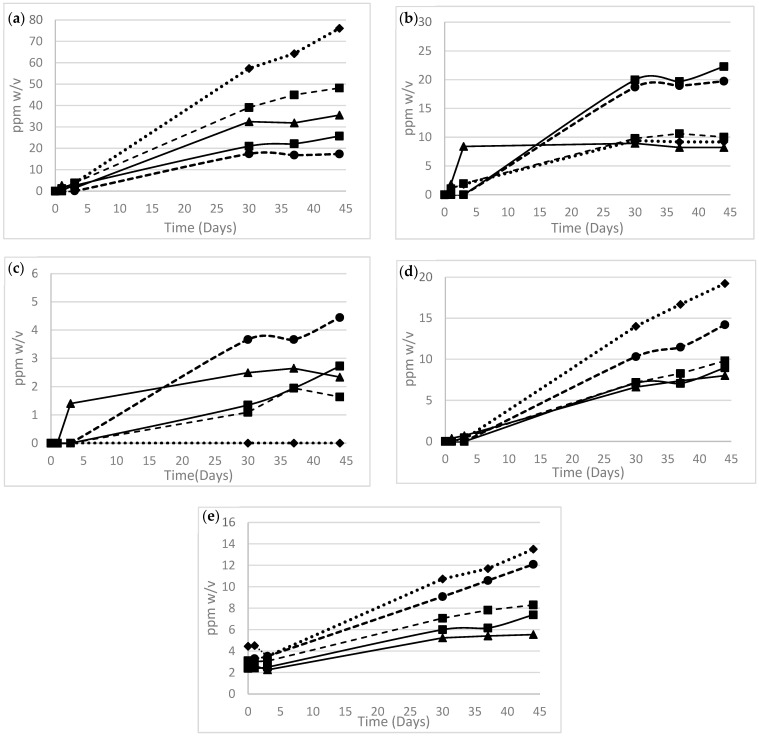
Concentrations of degradants found in simplified solutions; (**a**) 2,3-DHBA, (**b**) catechol, (**c**) muconic acid, (**d**) maleic acid, and (**e**) formic acid in simplified solution vs. time at pH 3.5 (-●-), 4 (■), 4.5 (▪◆▪), 5 (-■-), and 6 (▲). Note that the variation of the *y*-axis unit range is to aid clarity.

The evolution of the degradants in simplified solutions (Figure 7) was unsurprisingly faster than for the PEFs, likely owing to the micellation of formulation components and potential reactions of components of the flavoring element resulting in uncolored degradants. New signals in the aliphatic region evolving over time lend credence to this suggestion, but owing to the proprietary nature of the formulations, this will not be discussed further.

pH significantly influences degradant formation kinetics, showing non-uniform behavior linked to both degradant formation and subsequent reactions [28]. This aligns with previous literature demonstrating pH below 4 being optimal for the oxidation of salicylic species in uncatalyzed wet oxidations [29]. For instance, the highest 2,3-DHBA formation occurs at pH 4.5, while catechol formation/stabilization is favored at pH < 4. Muconic acid has the shortest lifespan among degradants, as its rapid conversion to maleic acid at pH 4.5 makes muconic acid undetectable. Although formic acid could result from oxalic acid (Figure 7), its low initial concentration, increasing over time, suggests that SA degradation alone is not the sole source of formic acid evolution.

### 3.2. Packaging Discoloration

In TiO_2_-supplemented HDPE bottles (HDPE (White)), noticeable discoloring of the internal packaging surface occurred rapidly at room temperature (2 weeks). Catechol, the source of solution discoloration, did not form appreciably under these conditions. Conversely, in HDPE without White pigmentation (HDPE (Natural)), PET, and glass packaging, no packaging discoloration was observed. However, as mentioned earlier, slight solution discoloration was noted for all formulations containing salicylic acid subjected to thermal treatment at 40–50 °C for 2–3 weeks or stored at 25 °C for 6–8 weeks. This suggests two mechanisms: one involving colored degradant formation and the other involving direct interactions between a component of the PEF and the packaging.

The surface discoloration specific to HDPE White packaging implicated the TiO_2_ pigment. The absence of discernible peaks in NMR spectra after several isopropyl alcohol extractions of the packaging (24 h incubation of packaging) indicated that this was not merely a loose association of degradants with the surface, rather, a specific chemical adsorption/reaction. Isopropyl alcohol was the only solvent used for extraction screening as it is standard procedure within an industrial environment to determine leaching from packaging into packaged formulations.

To investigate whether soluble TiO_2_ complexes could be contributing to the yellowing of the solution and surface of HDPE White, an ICP-MS analysis was conducted on proprietary formulations in both White and Natural HDPE. However, the concentrations of titanium determined did not significantly differ between packaging samples at any time or temperature of stress, as illustrated in Supporting Information Table S3. The fact that the Ti concentrations for both HDPE (White) and HDPE (Natural) are both very low supports the principal contention that the discoloration of the internal surface of the container is occurring by a direct reaction with Ti on the surface, not liberated Ti species in the formulation.

For HDPE (White), the extended TiO_2_ network means that only the surface could react with potential chelating species such as salicylic acid/maleic acid. Should they form, the complexes would remain on the surface and undissolved in the solution. The evolution of this distinctive yellow color over time on the surface of HDPE (White) appears to coincide with the formation in the solution of maleic acid, and the relative abundance of SA would make surface Ti complex formation by either specificity feasible. To evidence this surface reaction, fragments of discolored HDPE (White) compared with fresh HDPE (White) were analyzed by ATR-IR (Supporting Information Figure S3).

IR absorption bands at 1552 and 675 cm^−1^ are indicative of a metal carboxylate via the attenuated C=O vibrational stretch (sym) and M-O-C vibrational stretch (asym), respectively.

**Figure 8:**
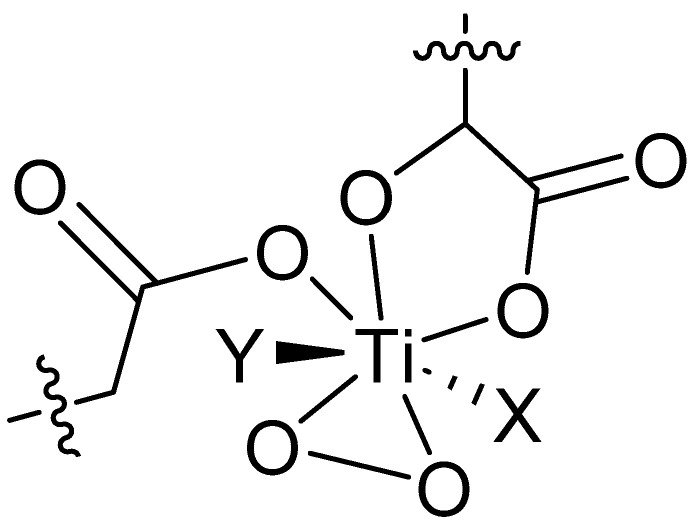
Proposed arrangement of surface titanium, activated by H_2_O_2_. Peroxo titanium complex with carboxylate and hydroxy carboxylate ligands. Adapted from [30]. The remaining two sites (X and Y) bonded to Ti come from two oxygen atoms of the oxo group bridging two further titanium atoms. This is represented below for both carboxylate and hydroxy carboxylate ligands, e.g., salicylic, citric, and acetic acids.

There is a literature precedent for the formation of titanium complexes, which react readily with carboxylic acids, such as SA and maleic acid, to give a range of colored compounds; however, they do not form spontaneously. TiO_2_ is a stable extended structure; however, as per the study by M. Kakihana et al., titanium oxide can be activated by peroxide ions [30]. The peroxo ion (O_2_)^2−^ binds to Ti in a h2-fashion, occupying two coordination sites, and (O_2_)^2−^ is bonded to the metal in a triangular bidentate manner (Figure 8).

The hypothesis posits that Ti(OH)_4_ or TiO_2_·xH_2_O, formed by the surface reaction of hydrogen peroxide from the solution, can form a highly coordinated surface peroxo-hydroxo titanium complex, represented as [Ti(O_2_)(OH)_3_]^−^. This complex subsequently creates colored surface chelates with salicylic acid (SA) and suitable oxidative degradants over time. Notably, both dicarboxylates and β-hydroxycarboxylic acids have been demonstrated to form yellow/orange complexes through this mechanism (Supporting Information Figure S4) [30]. It is important to note that this mechanism for discoloration cannot be proven based on the data, and potentially XRD and/or SEM data could be used to evidence this further.

## 4. Conclusions

**Figure 9:**
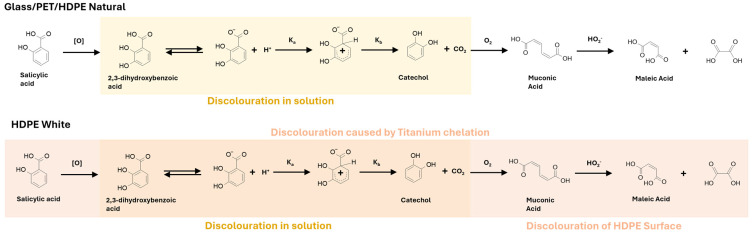
Discoloration contribution summary for packaging and component discoloration.

In summary, two distinct discoloration mechanisms have been suggested (Figure 9):

(1) HDPE (White) packaging discoloration: The potential ligation of peroxide-activated titanium (Ti) on the HDPE surface, by either SA or oxidative pathway degradants, may result in the formation of a colored surface layer. This activation of the TiO_2_ occurs via attack by H_2_O_2_. This Ti complexation process does not occur in glass, PET, or HDPE (Natural) due to the absence of TiO_2_.
(2) Discoloration in SA and peroxide formulations: The peroxide-mediated oxidation of SA leads to catechol formation through 2,3-dihydrobenzoic acid decarboxylation. Additionally, components of the proprietary flavoring in the formulation are oxidized at the expense of SA, explaining the difference in the oxidation rate of SA for proprietary vs. experimental formulations.

When developing commercial formulations, well-established APIs, solubilizing agents, acidity regulators, and other excipients are typically used to create viable products. However, these components can interact chemically and physically, resulting in undesirable consumer properties such as the odor, taste, texture, or appearance.

This work represents a novel approach by applying DOSY and 1D ^1^H NMR techniques to troubleshoot these formulation interactions quickly and effectively. This innovative method enabled multiple identifications, which significantly influenced the initial choice of packaging materials and the selection of excipients.

The novelty of our work lies in the practical application of these advanced analytical tools to improve product quality and enhance the overall consumer experience. This approach has the potential for broader applications beyond our specific study [31]. It can be adapted to address similar formulation challenges in plastic manufacturing, recycling, and re-filling processes. This approach not only facilitates the optimization of product stability, but also mitigates the inadvertent formation of problematic compounds, contributing to the development of sustainable and consumer-friendly solutions in the field.

## Data Availability

The datasets for this study are available on request.

## Supplementary Materials

The following supporting information can be downloaded at: https://www.mdpi.com/article/10.3390/pharmaceutics16030320/s1. Figure S1: NMR spectra 1D ^1^H of salicylic acid in H_2_O/D_2_O: (Top) aromatic peaks; (Bottom) Zoomed in with satellite peaks of aromatic peaks assigned; Figure S2: ^1^H NMR spectrum (zoomed) of (a) 2,3-dihydroxybenzoic acid (b) maleic acid (c) o-catechol and (d) the proprietary formulation in glass container 7 weeks at 40 °C; Figure S3: HDPE White (no discolouration) versus yellow surface analysis comparison with ATR-IR. Peaks that present for yellowed surface but are absent in non-discoloured surface are highlighted [12,13]; Figure S4: To visually demonstrate the rapid formation of colored compounds, hydrogen peroxide and SA were added to Ti(OMe)_4_, closely resembling the TiO_2_ system in HDPE White. This phenomenon was also observed with the oxidized derivative maleic acid, which exhibited a strong orange color when exposed to Titanium methoxide and hydrogen peroxide, indicating a similar coloration interaction with oxidized derivatives of salicylic acid. (Left to right) Ti(OMe)_4_ and H_2_O_2_; Ti(OMe)_4_ and H_2_O_2_ and SA; Ti(OMe)_4_ and SA within 1 min of combination; Table S1: Batch numbers and conditions for all samples analysed listed below in table; Table S2: Table of qNMR integral values over 24 h time range of for a representative PEF sample to show negligible change in component concentration whilst refrigerated; Table S3: ICP-MS data acquired for different packaging type PEFs at stored at 40 °C over 6 weeks.
