# Supplementary material for: Using Nuclear Magnetic Resonance to Troubleshoot a Stability Issue in a Real-World Formulation Chassis—Application to Consumer Oral Healthcare
Source: Pharmaceutics. 2024 Feb 24;16(3):320. doi: 10.3390/pharmaceutics16030320 (PMC10974615; doi:10.3390/pharmaceutics16030320)
Supplement: Supplementary file 1 [file pharmaceutics-16-00320-s001.zip › pharmaceutics-2856963-supplementary.pdf]

Using Nuclear Magnetic Resonance to Troubleshoot a Stability Issue in a Real-World Formulation Chassis—Application to Consumer Oral Healthcare

Tomris Coban [1], Hannah Sykes [2], Shreedhar Kulkarni [2], Robert A. Lucas [2], Cameron Robertson [1] and Adam Le Gresley [1,*]

[1] Life Sciences, Pharmacy and Chemistry, HSSCE Faculty, Kingston University, Kingston upon Thames KT1 2EE, UK; t.coban@kingston.ac.uk (T.C.); c.robertson@kingston.ac.uk (C.R.)
[2] Haleon PLC, Weybridge KT13 0DE, UK; hannah.i.sykes@haleon.com (H.S.); shreedhar.x.kulkarni@haleon.com (S.K.); robert.a.lucas@haleon.com (R.A.L.)
* Correspondence: a.legresley@kingston.ac.uk

Supporting Information

Table S1. Batch numbers and conditions for all samples analysed listed below in table.

Batch Number	Temperature/Pressure	Analysis Timeframe	Packaging	pH
**HALEON PLC1688B53**	15 °C/35psf	2 months	Glass	5.5
**HALEON PLC1688B53**	25°C/60psf	4 months	PET	5.5
**HALEON PLC1688B53**	40°C/75psf	4 months	PET	5.5
**HALEON PLC1688B53**	40°C/75psf	4 months	HDPE white	5.5
**HALEON PLC1688B53**	25°C/60psf	4 months	HDPE white	5.5
**HALEON PLC1688B54**	25°C/60psf	3 months	Glass	5.5
**HALEON PLC1688B55**	40°C/75psf	4 months	Glass	5.5
**HALEON PLC1688B56**	50°C/150psf	5 months	Glass	5.5
**HALEON PLC1688B58**	40°C/75psf	3 months	HDPE white + Refill Cap	5.5
**HALEON PLC1688B58**	40°C/75psf	1 month	PET + Refill Cap	5.5
**HALEONPLC 10317/76** no salicylic acid	40°C/75psf	1 month	HDPE White	3

**HALEONPLC 10317/87**	40ºC/75psf	0.5 months	Glass	3
**HALEONPLC 10317/80-3**	40ºC/75psf	3 months	PET	3
**HALEONPLC 10317/80**	40ºC/75psf	1 month	Glass	3
**HALEONPLC 10317/80**	40ºC/75psf	1 month	HDPE natural	3
**HALEONPLC 10317/80**	40ºC/75psf	1 month	HDPE white	3
**HALEONPLC 10317/80**	40ºC/75psf	1.5 months	PET	3
**HALEONPLC 10317/128**	40ºC/75psif	1 month	Glass	3
**HALEONPLC 10317/128**	40ºC/75psf	2 months	Glass	3
**HALEONPLC 10317/144** 2x concentration	40ºC/75psf	1.5 month	Glass	3
**New Cap extract** i-ProOH, evaporated to residue	15 ºC/35psf	N/A	HDPE white	5.5
**HDPE Bottle extract** i-ProOH, evaporated to residue	15 ºC/35psf	N/A	HDPE white	5.5
**Polypropylene** i-ProOH, evaporated to residue	15 ºC/35psf	N/A	Polypropylene	5.5
**PET Bottle extract** i-ProOH, evaporated to residue	15 ºC/35psf	N/A	PET	5.5
**KU_MM1_a** HDPE white token	40ºC/75psf	1 month	Glass	5.5
**KU_MM1_b**	40ºC/75psf	1 month	Glass	5.5
**KU_MM2_a** HDPE white token/no sal. acid	40ºC/75psf	1 month	Glass	5.5
**KU_MM2_b**	40ºC/75psf	1 month	Glass	5.5

no sal. acid				
**KU_MM3_a** HDPE white token/no sls	40°C/75psf	1 month	Glass	5.5
**KU_MM3_b** no sls	40°C/75psf	1 month	Glass	5.5
**KU_MM4_a** HDPE white token/no flavouring	40°C/75psf	1 month	Glass	5.5
**KU_MM4_b** No flavouring	40°C/75psf	1 month	Glass	5.5

Table S2. Table of qNMR integral values over 24 h time range of for a representative PEF sample to show negligible change in component concentration whilst refrigerated

GSKHALEON PLC1688B55	qNMR Absolute Integral values		
**Fridge time (hours)**	**Sal acid**	**Formic acid**	**DHBA**
0	481338213	15804234	1634711.3
2	481021952	15745255	1638120.6
24	481191099	15645537	1635186.6
**RSD**	0.03	0.51	0.11

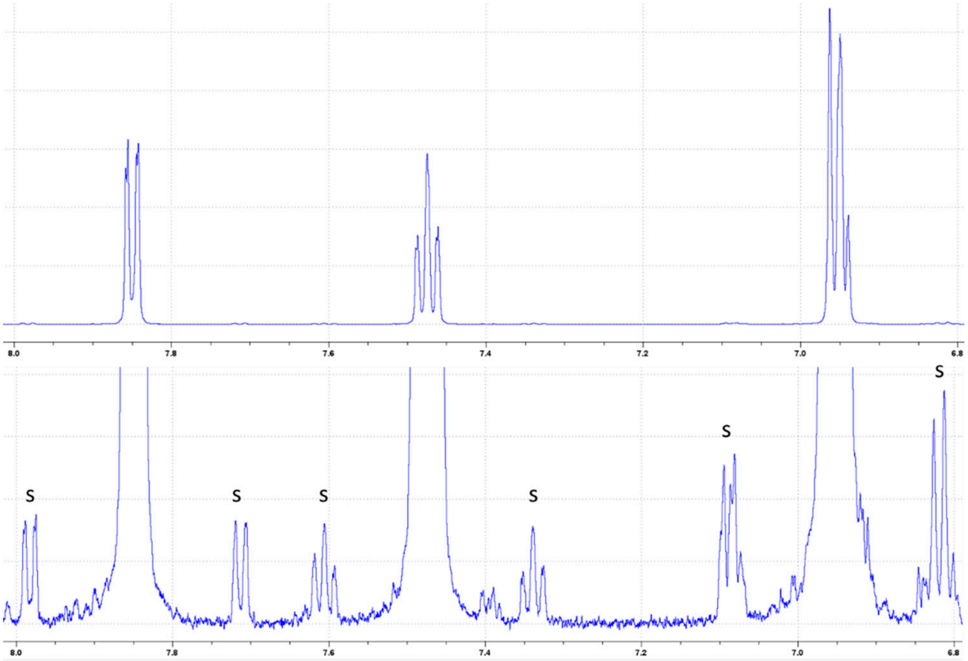

Figure S1. NMR spectra 1D ^{1}H of salicylic acid in H_2O/D_2O: (Top) aromatic peaks; (Bottom) Zoomed in with satellite peaks of aromatic peaks assigned.

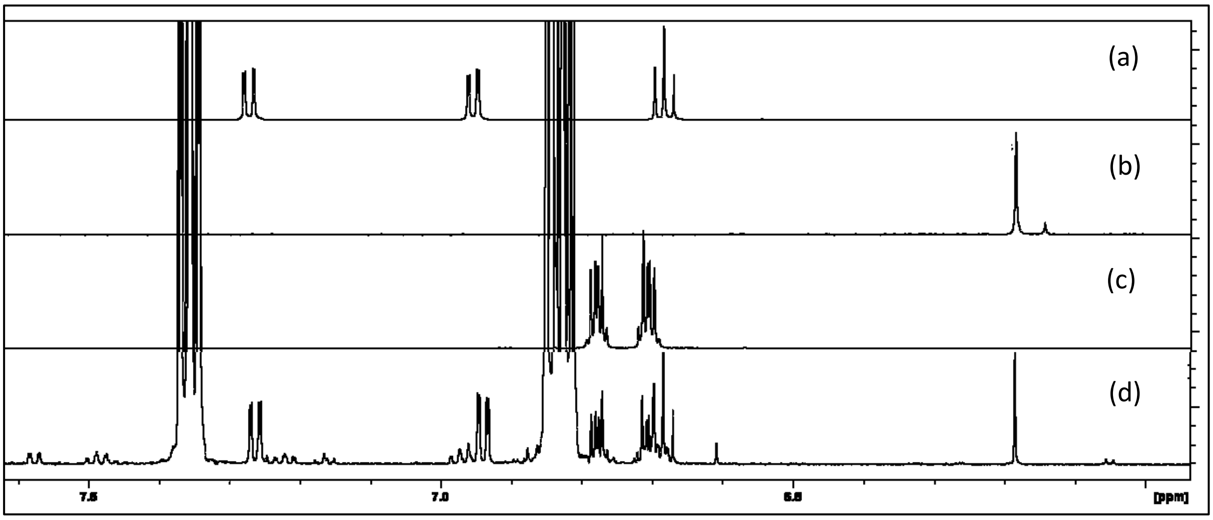

Figure S2. ^{1}H NMR spectrum (zoomed) of (a) 2,3-dihydroxybenzoic acid (b) maleic acid (c) o-catechol and (d) the proprietary formulation in glass container 7 weeks at 40°C.

Table S3. ICP-MS data acquired for different packaging type PEFs at stored at 40°C over 6 weeks.

Packaging	Ti conc. (ppb)	RSD
**HDPE White**	60.13	21.3
**HDPE natural**	59.09	15.9
**Glass**	0.09	8.7

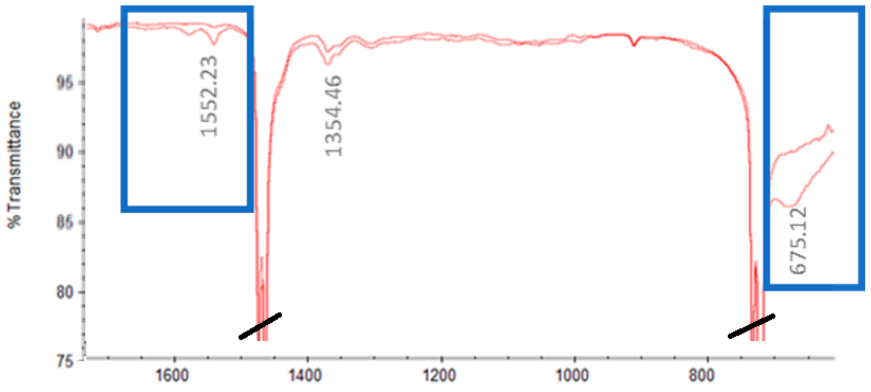

Figure S3. HDPE White (no discolouration) versus yellow surface analysis comparison with ATR-IR. Peaks that present for yellowed surface but are absent in non-discoloured surface are highlighted [12,13].

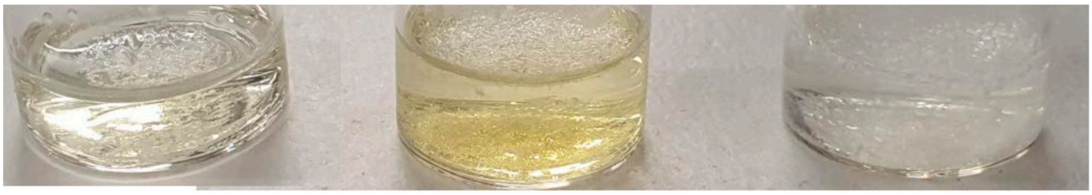

Figure S4. To visually demonstrate the rapid formation of colored compounds, hydrogen peroxide and SA were added to $Ti(OMe)_4$, closely resembling the TiO_2 system in HDPE White. This phenomenon was also observed with the oxidized derivative maleic acid, which exhibited a strong orange color when exposed to Titanium methoxide and hydrogen peroxide, indicating a similar coloration interaction with oxidized derivatives of salicylic acid. (Left to right) $Ti(OMe)_4$ and H_2O_2; $Ti(OMe)_4$ and H_2O_2 and SA; $Ti(OMe)_4$ and SA within 1 minute of combination.
